# Molecular prevalence and subtype distribution of *Blastocystis* spp. among children who have diarrheia or are asymptomatic in Wenzhou, Zhejiang Province, China[Fn FN1]

**DOI:** 10.1051/parasite/2024012

**Published:** 2024-03-06

**Authors:** Wei Zhao, Guangxu Ren, Long Wang, Lisha Xie, Jiayang Wang, Jialiang Mao, Yanbin Sun, Gang Lu, Huicong Huang

**Affiliations:** 1 Department of Parasitology, School of Basic Medical Sciences, Wenzhou Medical University Wenzhou Zhejiang 325035 China; 2 Department of Pathogenic Biology, Hainan Medical University, Haikou, Hainan, China; Hainan Medical University-The University of Hong Kong Joint Laboratory of Tropical Infectious Diseases, Hainan Medical University Haikou Hainan China; Key Laboratory of Tropical Translational Medicine of the Ministry of Education, Hainan Medical University Haikou 571199 China

**Keywords:** *Blastocystis*, Children, Genetic characteristics, Subtype, China

## Abstract

*Blastocystis* sp., a significant zoonotic parasite with a global distribution, was the focus of this study, which aimed to investigate its prevalence and genetic diversity among diarrheic and asymptomatic children in Wenzhou, China. We collected 1,032 fecal samples from Yuying Children’s Hospital, Wenzhou, China, comprising 684 from children with diarrhea and 348 from asymptomatic children. Genomic DNA extracted from these samples was used to detect *Blastocystis* spp. by PCR, targeting the small subunit ribosomal RNA gene. Subsequently, a phylogenetic tree was constructed, applying the maximum likelihood method. *Blastocystis* spp. were detected in 67 (6.5%) of the fecal samples. The prevalence rate of *Blastocystis* spp*.* in diarrheic children (8.8%; 60/684) was significantly higher than that in asymptomatic children (2.0%; 7/348) (*χ* ^2^ = 17.3, *p* < 0.001). Sequence analysis of the *SSU rRNA* gene identified five known *Blastocystis* spp. subtypes, ST1 (*n* = 12), ST2 (*n* = 5), ST3 (*n* = 35), ST4 (*n* = 12), and ST7 (*n* = 3). ST1 and ST3 were present in both diarrheic and asymptomatic children, while ST2, ST4, and ST7 were exclusive to diarrheic children. Intra-subtype genetic polymorphisms were identified, comprising four variations in ST1 (ST1-1 to ST1-4), five in ST3 (ST3-1 to ST3-5), two in ST4 (ST4-1 and ST4-2), and two in ST7 (ST7-1 and ST7-2). Notably, ST1-2 to ST1-4, ST3-3 to ST3-5, and ST7-1 and ST7-2 represent newly identified variations. The composition and genetic characteristics of subtypes among children in this region suggest various sources of infection, including human-to-human and animal-to-human transmission.

## Abstract (fr)

*Blastocystis* sp., un parasite zoonotique important avec une distribution mondiale, était au centre de cette étude, qui visait à étudier sa prévalence et sa diversité génétique parmi les enfants diarrhéiques et asymptomatiques de Wenzhou, en Chine. Nous avons collecté 1 032 échantillons fécaux à l’hôpital pour enfants Yuying de Wenzhou, en Chine, dont 684 provenant d’enfants souffrant de diarrhée et 348 d’enfants asymptomatiques. L’ADN génomique extrait de ces échantillons a été utilisé pour détecter *Blastocystis* sp. par PCR, ciblant le gène de la petite sous-unité de l’ARN ribosomal. Par la suite, un arbre phylogénétique a été construit, en appliquant la méthode du maximum de vraisemblance. *Blastocystis* sp. a été détecté dans 67 (6,5 %) des échantillons fécaux. Le taux de prévalence de *Blastocystis* spp. chez les enfants diarrhéiques (8,8 % ; 60 / 684) était significativement plus élevé que chez les enfants asymptomatiques (2,0 % ; 7 / 348) (*χ*^2^ = 17,3, *p* < 0,001). L’analyse de la séquence du gène de l’ARNr SSU a identifié cinq sous-types de *Blastocystis* spp., ST1 (*n* = 12), ST2 (*n* = 5), ST3 (*n* = 35), ST4 (*n* = 12) et ST7 (*n* = 3). Les sous-types ST1 et ST3 étaient présents chez les enfants diarrhéiques et asymptomatiques, tandis que ST2, ST4 et ST7 étaient exclusifs aux enfants diarrhéiques. Des polymorphismes génétiques intra-sous-types ont été identifiés, comprenant quatre variations dans ST1 (ST1-1 à ST1-4), cinq dans ST3 (ST3-1 à ST3-5), deux dans ST4 (ST4-1 et ST4-2) et deux dans ST7 (ST7-1 et ST7-2). Notamment, ST1-2 à ST1-4, ST3-3 à ST3-5, ST7-1 et ST7-2 représentent des variations nouvellement identifiées. La composition et les caractéristiques génétiques des sous-types chez les enfants de cette région suggèrent diverses sources d’infection, notamment la transmission interhumaine et animale.

## Introduction

*Blastocystis* sp., a eukaryotic parasite, is frequently reported in both immunocompetent and immunocompromized individuals. Due to the significant numbers of *Blastocystis* detection in both symptomatic and asymptomatic people, its pathogenic role is controversial. However, there is an increasing body of evidence suggesting that immunocompromized individuals are more susceptible to *Blastocystis* infection and suffer from associated symptoms such as gastrointestinal illnesses and/or urticaria. [10]. *Blastocystis* spp. infect a broad range of animal hosts and are primarily transmitted through the fecal-oral route, involving ingestion of contaminated water or food, contact with fecally contaminated environments, and direct interaction with infected hosts [2]. Furthermore, documented global waterborne blastocystosis outbreaks underscore the parasite’s significant public health implications [5]. Monitoring *Blastocystis* infections in populations to determine prevalence and identify accurate sources of infection is crucial for controlling *Blastocystis* outbreaks.

PCR-based molecular diagnostic methods are increasingly utilized to identify infection sources and assess cross-species transmission of *Blastocystis* spp. [28]. Genetic analysis of the small subunit ribosomal RNA gene (*SSU rRNA*) has revealed approximately 40 *Blastocystis* spp*.* subtypes [24, 40]. Humans can harbor subtypes ST1 to ST9, ST12 to ST14, ST16, ST35 and ST41, with over 90% of human *Blastocystis* strains belonging to ST1 to ST4 [21, 24]. Certain human subtypes are also found in animals, for example, ST3 in non-human primates, ST4 in rodents, ST5 in pigs, ST7 in birds, ST6 in sheep, and ST14 in cattle [2, 21]. Other subtypes are exclusive to specific animal species and are thus considered host-specific [2]. Therefore, understanding the distribution of subtypes in populations is crucial to infer potential sources of infection and transmission routes, aiding in the precise control of *Blastocystis* transmission.

Research on the molecular epidemiology of blastocystosis in humans and animals is increasingly active in China. To date, *Blastocystis* spp. infection has been reported in humans across at least 12 provinces and in more than 25 different animal hosts in China, with nine subtypes being identified in humans and 14 subtypes in animals [9, 19]. Because subtypes such as ST1, ST3, and ST5 have been identified in both animals and humans within the same province, this suggests possible zoonotic transmission between humans and animals [6]. However, data on *Blastocystis* infection in both animals and humans are scarce for the southeast coastal city of Wenzhou. This study focused on determining the prevalence and subtype distribution of *Blastocystis* in children from Wenzhou, Zhejiang Province.

## Methods

### Ethical approval and participant consent

Ethical approvals for the investigations were obtained from the Ethics Committees of Wenzhou Medical University (reference number SCILLSC-2021-01). All participants provided written informed consent which was obtained from their parents or guardians. Participants and their parents/guardians were informed about the study’s objectives and methods before obtaining consent.

### Sample collection

From March 2021 to January 2022, 1,032 children (<6 years of age) were recruited at Yuying Children’s Hospital, Wenzhou, China. Of these children, 348 were asymptomatic, and 684 exhibited symptoms of diarrhea (Table 1). Parents or guardians, under guidance, collected fecal samples using plastic fecal collectors, appropriately labeled with the date of collection, patient’s age, and sex. After collection, the samples were stored at 4 °C.

**Table 1:** Prevalence and subtype distribution of *Blastocystis* in diarrheic and asymptomatic children in Wenzhou, China.

Groups	Diarrhea		Non-diarrhea		Total	
Sex	No. Positive/No. examined (%)	*Blastocystis* subtypes (*n*)	No. Positive/No. examined (%)	*Blastocystis* subtypes (*n*)	No. Positive/No. examined (%)	*Blastocystis* subtypes (*n*)
Boy	39/376 (10.4)	ST3 (14); ST1 (6); ST4 (11); ST7 (3); ST2 (5)	4/195 (2.1)	ST3 (2); ST1 (2)	43/571 (7.5)	ST1 (8); ST2 (5); ST3 (16); ST4 (11); ST7 (3)
Girl	21/308 (6.8)	ST3 (16); ST1 (4); ST4 (1)	3/153 (2.0)	ST3 (3)	24/461 (5.2)	ST3 (19); ST1 (4); ST4 (1)
Total	60/684 (8.8)	ST1 (10); ST2 (5); ST3 (30); ST4 (12); ST7 (3)	7/348 (2.0)	ST1 (2); ST3 (5)	67/1032 (6.5)	ST1 (12); ST2 (5); ST3 (35); ST4 (12); ST7 (3)

### DNA extraction

Genomic DNA was extracted from each fecal sample (180–200 mg) using a QIAamp DNA Stool Mini Kit (QIAGEN, Hilden, Germany), following the manufacturer’s instructions. The DNA was then eluted in 200 μL of AE buffer and stored at −20 °C until PCR analysis.

### PCR amplification

For *Blastocystis* spp. detection, a 500 bp fragment of the *SSU rDNA* gene was amplified using primers fwd-seq: 5′-GGAGGTAGTGACAATAAATC-3′ and rev-seq: 5′-TGCTTTCGCACTTGTTCATC-3′, with PCR reaction conditions as described by Santin *et al*. [25]. TaKaRa Taq DNA Polymerase (TaKaRa Bio Inc., Tokyo, Japan) was employed for all PCR amplifications. Quality control was ensured by including negative controls in all PCR tests. The amplified PCR products were analyzed using 1.5% agarose gel electrophoresis, visualized with a Gel Doc EZ UV-gel imaging system (Bio-Rad Inc., Hercules, CA, USA), and stained using GelRed (Biotium Inc., Hayward, CA, USA) for enhanced visibility.

### Nucleotide sequencing and analysis

PCR products of interest were sequenced at Sangon Biotech Co., Ltd (Shanghai, China). Sequence accuracy was ensured through bidirectional sequencing and, where necessary, additional sequencing of PCR products. Edited and aligned sequences of each strand were processed using Clustal X v2.1 (http://www.clustal.org/) and DNASTAR Lasergene EditSeq v7.1.0 (http://www.dnastar.com/) tools. Reference sequences were obtained from GenBank.

### Phylogenetic analysis

A phylogenetic tree for nucleotide sequences was constructed using Mega 7, employing the maximum likelihood method. The phylogenetic tree’s reliability and statistical support for its topology were assessed using 1,000 bootstrap replicates. Evolutionary distances were computed employing the Tamura-3 parameter model (the Best DNA/Protein Models for our phylogenetic tree).

### Nucleotide sequence accession numbers

The nucleotide sequences of *Blastocystis* ST obtained in this study were deposited in the GenBank database under accession numbers OR936677 to OR936690.

### Statistical analyses

Data analysis was performed with SPSS version 22.0 (SPSS Inc., Chicago, IL, USA). The chi-square test (χ^2^) was utilized to compare the prevalence of *Blastocystis* spp. between diarrheic and asymptomatic groups and between boys and girls. A *p*-value < 0.05 was considered indicative of statistical significance.

## Results

### Infection rates of *Blastocystis* spp.

Sequence analysis confirmed that 67 of the 1,032 fecal samples tested positive for *Blastocystis* spp., comprising 2.0% (7/348) in asymptomatic children and 8.8% (60/684) in children with diarrhea (Table 1)., The prevalence rates of *Blastocystis* spp. differed significantly between children with diarrhea and asymptomatic children (χ^2^ = 17.3, *p* < 0.001). *Blastocystis* prevalence was 7.5% (43/571) in boys and 5.2% (24/461) in girls (Table 1), with no significant difference in prevalence between the sexes (χ^2^ = 2.27, *p* > 0.05). Among children with diarrhea, *Blastocystis* prevalence was 10.4% (39/376) in boys and 6.8% (21/308) in girls. In the asymptomatic group, *Blastocystis* prevalence was 2.1% (4/195) in boys and 2.0% (2/153) in girls. Statistical analysis indicated no significant difference in *Blastocystis* prevalence between boys and girls in paired comparisons (χ^2^ = 2.67 and 0.28; *p* > 0.05).

### Sequencing of PCR amplicons

All 67 PCR amplicons were sequenced to identify *Blastocystis* spp. subtypes. Nucleotide sequence analysis identified five known subtypes (ST1, ST2, ST3, ST4, and ST7), with no mixed infections detected. ST3 was the most prevalent subtype (52.2%; 35/67), followed by ST1 and ST4 (17.9%; 12/67, each), ST2 (7.5%; 5/67), and ST7 (4.5%; 3/67). ST1 and ST3 were found in both asymptomatic and diarrheic children, while ST2, ST4, and ST7 were exclusive to diarrheic children (Table 1). Genotypic composition varied between boys and girls: ST2 and ST7 were exclusive to boys, while ST1, ST3, and ST4 were present in both boys and girls (Table 1).

### *Blastocystis* subtype variants

**Figure 1:**
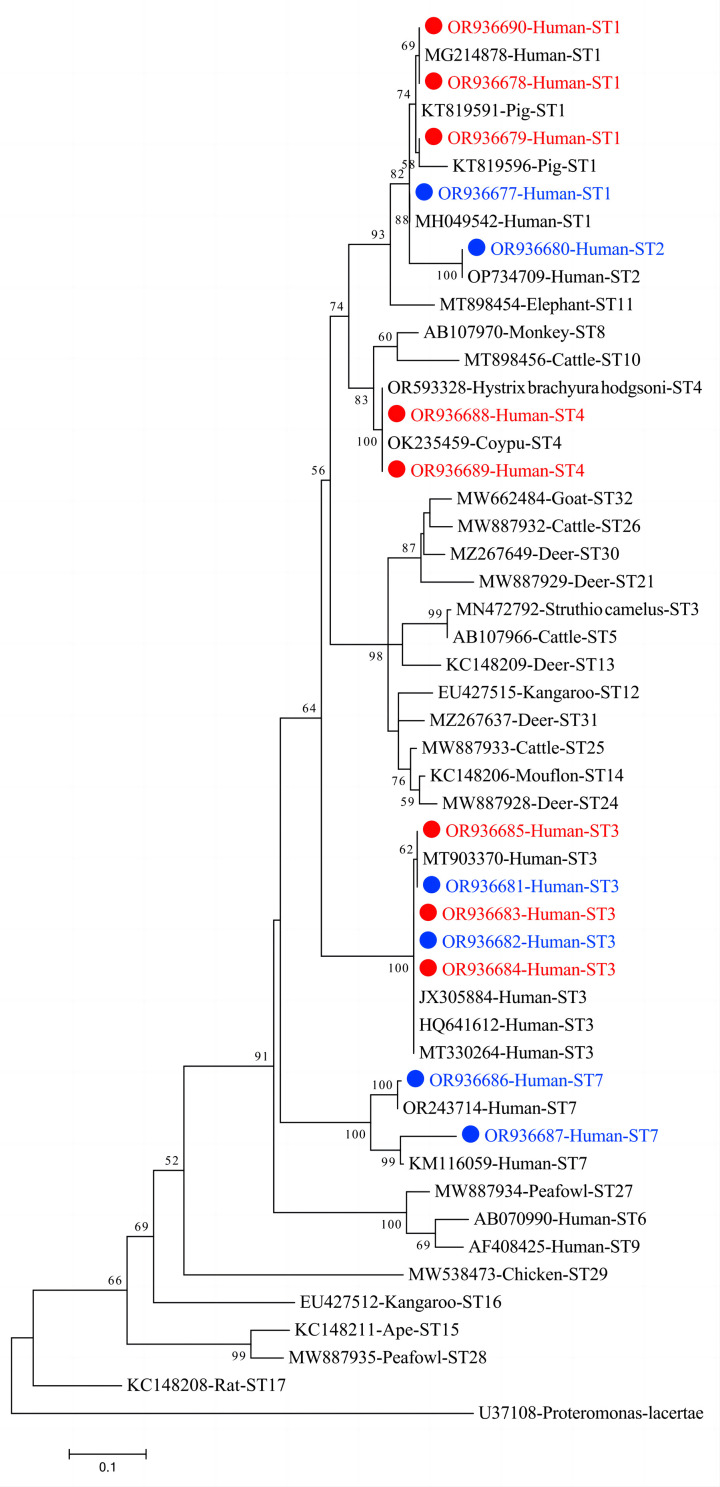
Phylogenetic tree of *Blastocystis* sp. subtypes based on *SSU rRNA* gene sequences. The phylogenetic relationships among different *Blastocystis* spp. STs were determined using Clustal X and Mega 7 software for genetic distance calculations and tree construction. The tree was constructed employing the maximum likelihood method, utilizing the Tamura-3 parameter model to calculate evolutionary distances. Positions containing gaps and missing data were excluded from the analysis. Bootstrap values were derived from 1,000 replicates to assess the reliability of the tree. In the tree, subtypes are denoted with blue and red fonts to distinguish between known and novel sequences identified in this study.

Similarity analysis of the 67 *Blastocystis* sequences yielded 14 representative sequences (OR936677–OR936690), comprising four from ST1, one from ST2, five from ST3, two from ST4, and one from ST7 isolates (Table 2). Of the four representative ST1 sequences, ST1-1 (OR936677) was previously described and shared 100% genetic identity with subtype ST1 MH049542 (*n* = 7). The other three, ST1-2 (OR936678) (*n* = 3), ST1-3 (OR936679) (*n* = 1), and ST1-4 (OR936690) (*n* = 1) have not been described previously and showed 99.28%, 99.77%, and 97.7% similarity to MG214878, KT819596 and KT819591, respectively. The five ST2 isolates shared a sequence (OR936680) with 100% similarity to the ST2 sequence (OP734709) from a human case in Mexico. The five representative ST3 sequences comprised ST3-1 (OR936681) (*n* = 19) and ST3-2 (OR936682) (*n* = 13), each displaying 100% genetic identity with the known subtypes ST3 MN472792 and MT330264, respectively. The remaining three, ST3-3 to ST3-5 (OR936683 to OR936685) (one each), previously undescribed, showed 99.55% similarity to MT903370 (human, South Korea), 99.77% to JX305884 (human, Colombia), and 99.77% to HQ641612 (human, Thailand), respectively. The two ST4 sequences, ST4-1 (OR936688) (*n* = 9) and ST4-2 (OR936689) (*n* = 3), were found to be identical to sequences from coypu in China (OK235459) and *Hystrix brachyura hodgsoni* in China (OR593328), respectively. Two novel ST7 sequences, ST7-1 (OR936686) (*n* = 2) and ST7-2 (OR936687) (*n* = 1), from the three ST7 samples were previously unreported, and share 99.77% and 99.75% similarity to the ST7 sequences derived from Chinese (OR243714) and Thai (KM116059) cases, respectively (Table 2). Overall, the nucleotide sequences of *Blastocystis* spp. subtypes from this study clustered with known subtypes in the phylogenetic tree (Figure 1).

**Table 2:** Similarity analysis of the *Blastocystis* sequences obtained in the present study.

Subtypes (*n*)	Accession no.	Ref. accession no.	Similarity	Host (country)
ST1-1 (7)	OR936677	MH049542	100%	Human (Malaysia)
ST1-2 (3)	OR936678	KT819596	99.28%	Pig (Thailand)
ST1-3 (1)	OR936679	MG214878	99.77%	Human (Poland)
ST1-4 (1)	OR936690	KT819591	97.97%	Pig (Thailand)
ST2 (5)	OR936680	OP734709	100%	Human (Mexico)
ST3-1 (19)	OR936681	MT330272	100%	Human (Thailand)
ST3-2 (13)	OR936682	MT330264	100%	Human (Thailand)
ST3-3 (1)	OR936683	HQ641612	99.77%	Human (Colombia)
ST3-4 (1)	OR936684	JX305884	99.77%	Human (Thailand)
ST3-5 (1)	OR936685	MT903370	99.55%	Human (South Korea)
ST4-1 (9)	OR936688	OR593328	100%	Asiatic brush-tailed porcupine (China)
ST4-2 (3)	OR936689	OK235459	100%	Coypu (China)
ST7-1 (2)	OR936686	OR243714	99.77%	Human (China)
ST7-2 (1)	OR936687	KM116059	99.75%	Human (Thailand)

## Discussion

A recent meta-analysis by Popruk *et al*., encompassing 92 studies from January 2002 to December 2019, revealed that *Blastocystis* prevalence in humans varies significantly by region: 41.0% in Africa, 53.8% in America, 52.0% in Australia and Oceania, 24.39% in Europe, and 21.6% in Asia, influenced by factors such as residence area, age, immune status, and diagnostic method [21]. Since the first reported case of *Blastocystis* infection in China in 1986, its prevalence has been observed across various provinces, ranging from 0.80% to 100%, with an overall infection rate of 3.37% [9, 19]. In this study, the infection rate of *Blastocystis* in children from Wenzhou was 6.5%, lower than in Jiangxi (35.9%) [39], Xinjiang (14.3%) [23], and Fujian (8.9%) [13], but higher than in Guangdong (5.0%) [34], Anhui (1.0%) [32], and Yunnan (3.1%) [38]. Overall, the infection rate of *Blastocystis* in Chinese children was lower compared to some developing countries, like Nigeria (84.0%) [22], Mexico (78.4%) [12], Thailand (49.3%) [33], Ecuador (39.2%) [30], and Indonesia (33.3%) [1]. These data indicate significant regional differences in *Blastocystis* infection rates.

Numerous factors influence the infection rate of *Blastocystis*, including the host’s immune status, underlying health conditions, and living environment [21]. The host immune system is closely linked to age, with children (<8 years old) having less mature immune functions than adults. Therefore, theoretically, children may have higher infection rates of *Blastocystis* than adults [4]. While some studies have observed statistical differences in infection rates between children and adults, others have reported higher rates in adults, as seen in individuals aged 18–39 years compared to children under 5 in Yunnan, Guangxi, and Heilongjiang provinces [19], indicating the multifaceted and complex nature of factors influencing *Blastocystis* infection rates.

This study found a significantly higher infection rate of *Blastocystis* in children with diarrhea compared to asymptomatic children, aligning with findings from previous reports [4, 16, 20, 38]. Zhang et al. reported that *Blastocystis* prevalence was higher in diarrheaic children (3.1%) compared to healthy controls (0.5%) in southwest China [38]. This indicates a potential relationship between *Blastocystis* infection and clinical symptoms like diarrhea. However, other studies report higher *Blastocystis* infection rates in asymptomatic populations, challenging this relationship [8]. Furthermore, the chosen detection method significantly influences *Blastocystis* detection rates [11]. Numerous studies indicate that molecular biology methods yield higher detection rates than morphological methods [26]. Therefore, adopting more precise detection methods is crucial to accurately assess the infection rate in the target population.

Poor hygiene practices significantly contribute to the high infection rate of *Blastocystis* [16]. Additionally, *Blastocystis* prevalence is higher in rural communities compared to urban settings [21]. *Blastocystis* is typically transmitted *via* the fecal-oral route, with increased risk factors including animal exposure, untreated water, and poor hand hygiene before meals [16]. Previous studies have documented significantly higher infection rates associated with the lack of hand washing after using the toilet [11]. Therefore, adherence to proper hygiene practices is a vital preventive measure against *Blastocystis* infection.

According to the review by Popruk *et al*. on the epidemiology and subtype distribution of *Blastocystis* in humans, subtypes ST1 to ST10, ST12, ST14, and ST16 have been identified in human populations [21]. With further research, these data are constantly updated, such as recent studies showing that ST35 and ST41 were also identified in humans [24]. Among these, subtypes ST1 to ST4 constitute 91.65% of all identified STs. ST3 is the most common, comprising 43.78%, followed by ST1 (27.22%), ST2 (14.75%), and ST4 (5.90%) [21]. Geographical variations are evident in the distribution of subtypes. ST4 is more prevalent in European countries, whereas ST2 is less common in Asia [21]. Regardless of the country, ST1 and ST3 consistently dominate. ST1 to ST7, ST12, and ST14 have been reported in the Chinese population, with ST3 (53.8%) and ST1 (36.5%) being the most prevalent. In China, mixed subtype infections, along with some unknown or novel subtypes, have been identified [9, 19]. In this study, five subtypes (ST1 to ST4 and ST7) were identified in children, with ST3 being the most common, aligning with findings from most studies in China [9]. Notably, a higher proportion of ST4 (17.9%) was observed compared to previous Asian studies (3.95%) [21].

The five known *Blastocystis* subtypes (ST1 to ST4 and ST7) identified in this study are also prevalent in various animals in China [18]: ST1 in non-human primates, dogs, foxes, goats, sheep, pigs, cattle, civets, bears, and birds; ST2 in non-human primates, bears, and some captive wild animals; ST3 in non-human primates, goats, sheep, cattle, pigs, raccoon dogs, and rex rabbits; ST4 in rodents, bears, and whooper swans; and ST7 in chickens, whooper swans, and some birds [7, 14, 15, 17, 29, 31, 35–37, 40, 41]. These findings suggest the potential for zoonotic transmission of *Blastocystis*. However, the epidemiological role of animals in *Blastocystis* transmission remains unclear due to the lack of data on local animal populations. Future studies should focus on collecting animal fecal specimens to explore this hypothesis, particularly from common domestic animals like pigs, cattle, sheep, and chickens, as well as wild rats near human dwellings.

Similarity analysis in this study revealed that 63.6% of ST1 and 91.4% of ST3 sequences correspond to previously described sequences, both originating from humans (Table 2). ST2 and ST4 sequences identified in this study match those previously described, with ST2 derived from humans and ST4 from rodents (Table 2). These findings indicate that childhood *Blastocystis* infection likely originates from humans and rodents. Additionally, this study uncovered novel gene sequences with subtle variations. Eight of the 13 representative sequences obtained were previously unreported (Table 2). The newly identified gene sequences may exhibit regional differences or originate from diverse infection sources. Future research should investigate potential animal hosts in this region to accurately evaluate the sources of these new sequences, using sequence homology analysis to trace the infection source.

Extensive research has been conducted on the association between *Blastocystis* subtypes and clinical symptoms. Previous studies have consistently shown the predominance of the ST3 subtype in patients with chronic gastrointestinal diseases [21]. Conversely, the ST4 subtype is predominantly found in patients with hematological malignancies. Additionally, a higher prevalence of *Blastocystis* ST4 has been observed in patients with chronic diarrhea, inflammatory bowel disease, and irritable bowel syndrome [27]. Popruk *et al*. reported that ST1 is often associated with asymptomatic infections, while ST4 is more commonly linked to symptomatic infections [21]. This study identified ST1 and ST3 subtypes in both diarrheic and asymptomatic children, whereas ST2, ST4, and ST7 were exclusive to children with diarrhea, indicating that the virulence of *Blastocystis* may vary among different STs. Different *Blastocystis* subtypes are closely correlated with variations in gut microbiota richness and diversity, as well as with distinct clinical conditions [3, 10]. Therefore, a thorough investigation into the distribution and impact of *Blastocystis* subtypes is crucial for understanding their role and significance in the human gut microbiome.

## Conclusions

This study presents the first detailed investigation into the infection rates and subtype distribution of *Blastocystis* in diarrheic and asymptomatic children in Wenzhou, Zhejiang Province, China. Results indicate an average infection rate of 6.5% in children, with a higher rate of 8.8% in those with diarrhea, significantly surpassing the 2.0% rate in non-diarrheic children. Five commonly known subtypes (ST1 to ST4 and ST7) were identified in this study. ST3 was the most prevalent (53.7%), demonstrating extensive variation within the subtype. Furthermore, ST2, ST4, and ST7 were exclusive to children with diarrhea, whereas ST1 and ST3 were found in both diarrheic and asymptomatic children. Similarity analysis suggests that ST1, ST2, and ST3 likely originate from human-to-human transmission, while ST4 may derive from rodents. The newly identified gene sequences may exhibit regional differences or stem from various infection sources. Future efforts should focus on intensifying molecular epidemiological investigations of *Blastocystis* in this region and others to more accurately assess the prevalence and public health significance of *Blastocystis* infections. This study not only improved our understanding of *Blastocystis* infection but also aided in evaluating the impact and future direction of public health interventions in the region.
